# Quality of Life after Cataract Surgery

**DOI:** 10.3390/jcm13175209

**Published:** 2024-09-02

**Authors:** Klaudia Błachnio, Aleksandra Dusińska, Julia Szymonik, Jan Juzwiszyn, Monika Bestecka, Mariusz Chabowski

**Affiliations:** 1 Student Research Club No. 180, Faculty of Medicine, Wroclaw Medical University, 50-367 Wrocław, Poland; klaudia.blachnio1@gmail.com (K.B.); oladusinska1@gmail.com (A.D.); julkas765@gmail.com (J.S.); 2 Department of Nursing and Obstetrics, Division of Anesthesiological and Surgical Nursing, Faculty of Health Science, Wroclaw Medical University, 51-618 Wrocław, Poland; jan.juzwiszyn@umw.edu.pl (J.J.); monbes@wp.pl (M.B.); 3 Department of Surgery, 4th Military Clinical Hospital, 5 Weigla Street, 50-981 Wrocław, Poland; 4 Department of Clinical Surgical Sciences, Faculty of Medicine, Wroclaw University of Science and Technology, 50-556 Wroclaw, Poland

**Keywords:** quality of life, Acceptance of Illness Scale, cataract, surgery, WHOQOL-BREF questionnaire

## Abstract

**Background:** The impact of medical intervention on a patient’s quality of life (QoL) is more and more important. Treatment success is defined not only in terms of the success of the procedure performed but also with regard to its impact on different areas of the patient’s life. The aim of the study was to assess the QoL of patients after cataract surgery and identify factors that affect it. **Methods:** Between January and March 2018, a survey was carried out among 100 patients who had undergone cataract surgery with intraocular lens implantation at the ‘Spektrum’ Clinical Ophthalmology Centre in Wrocław. The World Health Organization Quality of Life—BREF (WHOQOL-BREF) questionnaire and Illness Acceptance Scale (AIS) were used. **Results**: Most respondents (67%) rated their overall health as very good. The median score on the AIS was 34 (31.5–39), indicating a high level of illness acceptance. There was no statistically significant relationship (*p* > 0.05) between sex and QoL nor between the level of illness acceptance and QoL. We found no statistically significant relationships between place of residence and QoL (*p* > 0.05) nor between place of residence and AIS. **Conclusions:** The respondents reported the highest QoL scores for the environment domain and the lowest QoL scores for the social relationships domain. QoL had a positive impact on illness acceptance among the study patients. Younger patients (aged 50 or under) reported significantly higher scores for all the domains of QoL. Being employed was found to be associated with better QoL and greater illness acceptance.

## 1. Introduction

The National Eye Institute (NEI) defines a cataract as ‘a cloudy area in the lens of your eye that affects vision’ [1]. Globally, more than 90 million people are blind or visually impaired, and cataracts remain the leading cause of blindness in the world [2]. The risk of cataracts has been associated with such factors as age, sex, race, level of education, refraction, diabetes, smoking, change in bodyweight and multivitamin use [3]. In developed countries, the prevalence of cataracts is correlated with population aging, and they are more common in people aged over 65 [4].

Many people with a cataract do not know they have it, as the condition is insidious, painless and slow to progress. Significant symptoms may not appear until the cataract is well developed [5]. As noted by Gupta et al., vision loss inevitably results in impaired quality of life (QoL) [6]. There are several types of cataracts, which have different symptoms. This is due to the different ways in which a clouding of the lens affects the transmission of light [7]. The most common type of cataract is senile cataracts, which develop as a result of natural changes in the lens of the eye. Traumatic cataracts can develop as a result of damage to the eye, and certain types of radiation can cause cataracts, which we then call radiation cataracts. Children are born with cataracts or acquire them after birth—both types are called pediatric cataracts. After cataract surgery, it is possible for scar tissue to form in the eye, which can cause vision to become cloudy again. This is known as secondary cataract [1]. Cataracts are also classified based on their location within the lens. Nuclear cataracts occur at the lens’ center, where the nucleus gradually darkens with age, transitioning from clear to yellow and occasionally brown. Cortical cataracts affect the layer surrounding the nucleus, appearing as wedge-shaped or spoke-like opacities. Posterior capsular cataracts develop in the outer rear layer of the lens, often progressing more rapidly than other types [8]. Nevertheless, Berrios et al. suggest that the QoL of cataract patients does not differ significantly depending on the type of cataract they have [9]. No matter its type, a cataract commonly leads to visual acuity loss, which may progress gradually [7]. Cataract patients also report such symptoms as halos around lights and glare in the field of vision [7]. Other symptoms, such as increased sensitivity to light or fading colors, occur depending on the location of the cloudy areas in the lens. Cataract patients may also experience double vision in one eye due to local changes in the refractive index [7,10]. As noted by Holloway et al., impaired visual function limits the patient’s independence [11]. Given the whole clinical picture of the condition, an untreated cataract leads to reduced QoL and life expectancy [12].

Until recently, treatment success was defined only in terms of medical criteria. Currently, the impact of medical intervention on the patient’s quality of life is also taken into account [13]. The World Health Organization (WHO) defines QoL as ‘an individual’s perception of their position in life in the context of the culture and value systems in which they live and in relation to their goals, expectations, standards and concerns’ [14]. QoL is a particularly important parameter, as it is an indicator of treatment success and a measure of the patient’s satisfaction with life overall [15]. With this approach, treatment success is defined not only in terms of the success of the procedure performed but also with regard to its impact on different areas of the patient’s life [16]. It is crucial to understand it and take it into account when developing treatment plans in order to effectively improve the well-being of patients [16].

QoL is difficult to measure due to the subjectivity of an individual’s experiences. However, there are tools (scales and survey questionnaires) which can be used to collect data and arrange them appropriately [17]. The term ‘vision-related QoL’ (VR-QoL) covers the multifactorial impact of visual function on an individual’s overall well-being. The concept covers not only the measurable aspects of visual impairment, such as difficulties in performing vision-related tasks, but also its consequences for emotional well-being and social relations. Moreover, factors affecting VR-QoL include comfort and satisfaction with visual function, which underscores the complex interaction between visual acuity and overall QoL [18].

The QoL of patients with cataracts can be measured by evaluating their ability to perform daily activities and by assessing their level of comfort and satisfaction with life after surgery [19,20,21]. Improved QoL is one of the main aims of treatment in patients with cataracts. Therefore, it is necessary to carry out regular research to assess the QoL of cataract surgery patients, as it may help improve not only procedures directly related to the treatment process but also perioperative and long-term care.

Thus, the aim of the study was to assess the QoL of patients after cataract surgery and identify factors that affect it.

## 2. Material and Methods

Between January and March 2018, a co-author of the present study (M.B.) carried out a survey among 100 patients who had undergone cataract surgery with intraocular lens implantation at the ‘Spektrum’ Clinical Ophthalmology Centre in Wrocław. Surveys were conducted among patients who had cataract surgery at least one year previously. The patients in the study underwent surgery for age-related cataracts. They were diagnosed with cortical cataracts based on the location of the lens opacity. In all cases, cataract surgery was carried out using phacoemulsification. The participants had cataract surgery for one eye, and they were day-case patients. All respondents provided informed consent to participate in the study and answered in writing the questions included in the study questionnaires. The study was approved by the Bioethics Committee of the Wrocław Medical University (no. KB-9/2018). Participation in the study was anonymous and voluntary.

The participants included women (61%) and men (39%). Their mean age was 61.72 years (SD = 11.43). The majority of participants lived in urban areas (75%) with their families (67%). Most of them were in a relationship (58%), had secondary education (55%) and were employed (55%). A total of 52% of respondents had been diagnosed with a cataract in the 5 years before surgery, 35% more than 5 years prior to surgery, and only 13% had been diagnosed in the year before surgery. Regarding comorbidities, most respondents had hypertension (60%) and did not have diabetes (70%) or glaucoma (94%).

We assessed the QoL of the respondents using the WHOQOL-BREF questionnaire, which is a popular, validated quality of life assessment instrument developed by the WHO. The WHOQOL-BREF produces scores for four domains of QoL: physical health, psychological health, social relationships and environment. The WHOQOL-BREF questionnaire comprises 26 items, scored on a five-point scale. The WHOQoL instrument utilizes a 0–100 scoring system, where higher scores correspond to better quality of life in a particular domain. Notably, research has established that a cut-off score of less than 60 for overall QoL demonstrates excellent sensitivity and negative predictive value in identifying individuals who are likely to experience poorer quality of life and dissatisfaction with their health status [22]. We also used the standardized Illness Acceptance Scale (AIS), which is intended for use in individuals with chronic conditions. AIS consists of eight statements describing the negative consequences of poor health scored on a five-point Likert scale. AIS is a cognitive-behavioral assessment tool that specifically targets the emotional and psychological dimensions of illness acceptance, allowing patients to self-evaluate their coping mechanisms and emotional responses to their disease. Notably, the AIS has demonstrated robust psychometric properties, including a high internal reliability coefficient of 0.82 (Cronbach’s alpha) and a test–retest reliability coefficient of 0.69 over a 7-month period, indicating its reliability and consistency in measuring patients’ perceptions of their illness [23]. AIS scores are usually stratified into three levels: scores of 8–15 indicate a low level of illness acceptance, scores of 16–28 indicate a moderate level of acceptance and scores of 29–40 indicate a high level of acceptance. In addition, we used our own questionnaires developed based on the National Eye Institute Visual Function Questionnaire (NEI VFQ-25), which is used to assess visual function in a broad sense. The questionnaires included questions concerning difficulties in performing daily activities, overall health and personal experiences of visual impairment.

Statistical analysis was carried out using R 4.4.1. Statistical significance was set at *p* < 0.05. The normality of distribution of the variables analyzed was assessed using the Shapiro–Wilk test. The test showed that the distribution of the variables was not normal. Therefore, correlations between variables were analyzed using the Spearman’s correlation coefficient. The variables in two groups were compared using the Mann–Whitney test. The variables in three or more groups were compared using the Kruskal–Wallis test and, where there were statistically significant differences between the groups, a post-hoc Dunn’s test. Multiple linear regression was employed to model the potential impact of predictors on a quantitative variable. The regression parameters, alongside the 95% confidence intervals, were presented.

## 3. Results

Most respondents (67%) rated their overall health as very good, while only 1% rated their overall health as poor. The vast majority of respondents (80%) were satisfied with their vision. A total of 75% of respondents stated that their vision problems did not affect their daily functioning, whereas 25% stated that their vision problems limited their daily functioning (every day—16%, once a week—6%, three times a week—1%, once a month—2%, less often—1%). The most common difficulties reported by the participants were difficulties reading books and newspapers (21%), writing (11%), reading (6%), watching TV (6%) and using stairs (6%). A total of 77% of respondents had a driving license and 57% were drivers. Regarding the consequences of visual impairment, the largest proportions of respondents stated that they stayed home most of the time (35%) or felt anxious more often (31%) due to their vision problems. A total of 52% of respondents did not have any vision problems. Of the respondents who reported vision problems, 25% described them as minor, 22% described them as moderate and 1% described them as severe. A total of 99% of respondents reported that they visit their ophthalmologist to monitor eye health (37% of them visit their ophthalmologist less than once a year, 49% do so once year, 13% do so twice a year and 1% do so once a month).

The respondents reported the highest scores for the environment domain of QoL and the lowest scores for the social relationships domain. The median score on the AIS was 34, indicating a high level of illness acceptance (Table 1).

There was a significant (*p* ˂ 0.05) positive (r > 0) correlation between the level of illness acceptance and scores for the physical health domain of QoL. There was also a significant (*p* ˂ 0.05) positive (r > 0) correlation between the level of illness acceptance and scores for the psychological health domain of QoL. We also found a significant (*p* ˂ 0.05) positive (r > 0) correlation between the level of illness acceptance and scores for the social relationships domain of QoL. Moreover, there was a significant (*p* ˂ 0.05) positive (r > 0) correlation between the level of illness acceptance and scores for the environment domain of QoL (Table 2).

Respondents aged 50 or under reported significantly higher scores for the physical health domain compared to respondents aged 51–60, respondents aged over 70 and those aged 61–70.

Respondents aged 50 or under reported significantly higher scores for the psychological health domain compared to respondents aged 51–60, respondents aged 61–70 and those aged over 70. Respondents aged 51–60 reported significantly higher scores for the psychological health domain than respondents aged over 70.

Respondents aged 50 or under reported significantly higher scores for the social relationships domain compared to respondents aged 51–60, who reported significantly higher scores for this domain than respondents aged 61–70 and those aged over 70. Respondents aged 50 or under reported significantly higher scores for the environment domain compared to respondents aged 51–60, who reported significantly higher scores for this domain than respondents aged 61–70 and those aged over 70 (Table 3). The age groups were similar in size.

There was no statistically significant relationship (*p* > 0.05) between sex and QoL in the physical health (*p* = 0.150), psychological health (*p* = 0.199), social relationships (*p* = 0.529) and environment (*p* = 0.667) domains, and there was no statistically significant relationship between sex and the level of illness acceptance (Table 4).

Employed respondents reported significantly better QoL in the physical health domain (*p* < 0.001), psychological health domain (*p* = 0.006), social relationships domain (*p* < 0.001) and environment domain (*p* < 0.001) compared to unemployed respondents. Employed respondents also reported significantly higher levels of illness acceptance (*p* < 0.001) (Table 5).

We found a statistically significant relationship between place of residence and QoL in the social relationships domain (*p* = 0.022). We found no statistically significant relationships between place of residence and scores for the physical health, psychological health and environment domains of QoL (*p* > 0.05) and scores on the AIS (Table 6).

The multivariate linear regression model showed that each additional point of acceptance of illness increases the quality of life in the physical health domain by 0.178 points on average. The lack of employment decreases quality of life in this domain by an average of 2.178 points in relation to having permanent employment (Table 7).

The multivariate linear regression model showed that each additional point of acceptance of illness increases quality of life in the psychological health domain by 0.128 points on average. Having an age from 61 to 70 decreases quality of life in this domain by an average of 1.759 points compared to having an age of up to 50. Having an age over 70 decreases quality of life in this domain by an average of 2.441 points compared to having an age up to 50 (Table 8).

The multivariate linear regression model showed that each additional point of acceptance of illness raises quality of life in the social relationship domain by an average of 0.104 points. Having an age from 61 to 70 decreases the quality of life in this domain by an average of 1.813 points compared to having an age up to 50. Having an age over 70 decreases quality of life in this domain by an average of 2.215 points compared to having an age up to 50 (Table 9).

The multivariate linear regression model showed that each additional point of acceptance of illness raises quality of life in the environment domain by an average of 0.4 points. Having an age from 51 to 60 decreases quality of life in this domain by an average of 2.466 points compared to having an age up to 50. Having an age from 61 to 70 decreases quality of life by an average of 3.779 points compared to having an age up to 50. Having an age over 70 decreases quality of life in this domain by an average of 3.842 points compared to having an age up to 50 (Table 10).

## 4. Discussion

The importance of breakthroughs in cataract surgery has been consistently highlighted in the literature on the subject over the last ten years or so [24]. As noted by Thompson et al., cataract surgery with intraocular lens (IOL) implantation is currently considered to be the most effective and cost-effective [25] surgical procedure [7]. Today, cataracts are removed by small-incision surgery using advanced lens implants, which enable the combined treatment of a cataract and presbyopia or astigmatism, or both [12]. Such procedures make it possible to treat the problems experienced by the patient comprehensively, which seems to have an important impact on their QoL. Moreover, they provide a fast visual recovery and are associated with minimal complications [12]. Olson envisions that even more advanced lenses will be used in cataract surgery in the future as standard practice [24]. Therefore, it seems important to monitor the impact of these advances using questionnaires designed to assess QoL after cataract surgery.

The present retrospective study including patients after cataract surgery showed a significant positive correlation between cataract surgery and improved QoL. In particular, the study showed an association between cataract surgery and improved mental and emotional well-being and greater illness acceptance. The results suggest that cataract surgery may have an impact on the patient’s overall health-related QoL that extends beyond mere improvement in visual acuity. These findings have been confirmed by a number of other studies using different methods and questionnaires. In a study by Heemraz et al., cataract surgery patients reported a significant improvement in QoL even a few weeks after surgery [26]. A multi-cohort study by Queirós et al. showed that cataract surgery results in improved visual acuity, provides very good visual outcomes and is associated with minimal complications, thus having a positive impact on the patient’s QoL [27]. A study by Kuntorini et al. demonstrated that the QoL of patients after the first cataract surgery is similar to that of individuals with normal vision [28].

A number of factors contribute to improved QoL in patients after cataract surgery. As stressed by Gupta et al., the key factor is the significant improvement in the patients’ ability to carry out daily activities [6]. The present study showed that an increased ability to drive improved the QoL of the patients surveyed. A study by Signes-Soler et al. found a correlation between postoperative QoL and improved visual acuity [29], which is consistent with the findings from a study by Makabe et al., which demonstrated a relationship between an improvement in VR-QoL after cataract surgery and low preoperative VR-QoL and best-corrected visual acuity (BCVA) [30]. Interestingly, Miura et al. identified the length of the ellipsoid zone (EZ) of the photoreceptors as a factor that is significantly correlated with postoperative VR-QoL [31].

The participants of previous study had cataract surgery for one eye, and they had operation in an ophthalmologically specialized hospital located in a provincial city. Heemraz et al. found that the benefits of a second eye surgery are similar to those of the first eye surgery [26]. Studies have also demonstrated a relationship between where cataract surgery is carried out and postoperative QoL. A study by Xiang et al. showed that cataract surgeries carried out in district/county hospitals as part of blindness prevention programs significantly improved the QoL of the patients operated on but underperformed as compared to surgeries performed in a tertiary teaching hospital [31].

Several authors have investigated the impact of cataract surgery on the QoL of patients with comorbidities [31,32,33,34,35]. One of them demonstrated that VR-QoL in cataract patients with myopia is significantly impaired and improves significantly after cataract surgery. The improvement is greater compared to that observed in cataract patients with normal axial lengths [32]. It has also been shown that cataract surgery significantly improves QoL in patients with retinitis pigmentosa (RP) [30] and severe visual impairment due to age-related macular degeneration (AMD) [33]. Moreover, studies by Habash et al. and Yuasa et al. showed that the QoL of patients with glaucoma improves significantly after combined cataract and minimally invasive glaucoma surgery (MIGS) or cataract surgery alone, with greater improvement observed in patients undergoing combined cataract and MIGS surgery [34,35]. Furthermore, in a study by Li et al. including individuals who underwent cataract surgery, monocular patients reported a greater improvement in VR-QoL and greater reduction in the level of depression and anxiety compared to binocular controls. The authors of the study argue that cataract surgery should not be delayed in monocular patients [36].

Some authors also evaluate the quality of life of patients waiting for cataract surgery. Such pre- and post-operative comparisons seem to directly reflect the impact of the surgical procedure on patient functioning. In our study, the most common difficulties reported by participants were reading books and newspapers and writing. Similarly, in a study conducted by Oleś et al. among pre-operative patients, the greatest difficulties were also related to reading small print, newspapers and prices, with minimal improvement in these domains post-operatively, consistent with our finding that patients still frequently complain about these issues after cataract surgery. In our study, only 6% of respondents reported problems with watching television. In contrast, in the study by Oleś et al., the patients already reported average/low difficulty with watching television before cataract surgery [37]. A study conducted by Alias et al. showed that after undergoing surgery with a standard lens, patients reported improvements in visual function, mainly in far vision. The addition of an enhanced lens provides better performance in intermediate vision [38]. Amedo et al. observed a statistically significant improvement (*p* = 0.002) in the NEI VFQ-25 questionnaire scores, with a mean score of 62.58 ± 7.23 before surgery and one of 81.06 ± 8.47 after surgery. The VRQOL subscale scores were lowest in the domain of general health before surgery, with a mean score of 44.74 ± 13.50, but increased above 60 after surgery [39], consistent with our finding that patients generally reported their overall health as very good. The study also showed that all participants who reported an inability to perform certain daily activities due to cataract were able to resume these activities two months after surgery [39]. Another study found that the improvement in visual acuity after surgery was greater than the increase in quality of life (92.7% vs. 85.8%) [40].

The present study did not find a statistically significant relationship between sex and QoL after cataract surgery. However, the findings from studies carried out among different ethnical groups and in different geographical locations vary. Consistent with the present study, both a study by Heemraz et al. including patients of an ophthalmology department in London and a study by Kuntorini et al. in Indonesia showed no relationship between sex and QoL after cataract surgery [26,28].

Sex differences in QoL after cataract surgery reflect the degree of inequality in access to cataract surgery services in different regions of the world. Ye et al. noted in their study that female sex remains a significant barrier for access to cataract surgery services in South Asia [41]. The elimination of sex disparities in access to cataract surgery could reduce visual impairment, including blindness, attributed to untreated cataract by approximately 6% [41]. A study including patients who were provided with cataract surgery for free under a project designed to bridge the gap in access to cataract surgery between poor and non-poor people in Tangshan showed that male patients reported better QoL after cataract surgery than female patients [42]. In a study including Pakistani cataract patients who underwent cataract surgery, sex was found to be the only significant independent predictor of visual outcomes. Female patients were approximately four times more likely to have suboptimal visual acuity outcomes [43].

Studies have also shown differences in preoperative QoL between male and female cataract patients. In a study by Zhao et al. including cataract patients with and without previous cataract surgery experience, older age and female sex were found to be associated with a more severe impairment of cognitive function [44]. A study by Sharma et al. in India showed that female cataract surgery patients have a serious visual impairment in the better eye at the time of surgery and tend to choose a less expensive surgery option, which may correlate with a lower postoperative VR-QoL [45]. A study by López Sánchez et al. among Spanish patients with diabetes found a significant association between the presence of cataracts and significantly higher odds of depression and chronic anxiety in female diabetic patients. No such association was found for male patients [46]. In a study by Partyka-Mizeracka et al. among Polish cataract patients after cataract surgery, female patients rated their QoL and health slightly better than male patients [47].

The aforementioned findings suggest that the sex differences in QoL after cataract surgery may be due to cultural and socioeconomic factors as well as factors relating to access to healthcare. Further research is needed to better understand the complex interactions between these factors and design strategies to eliminate sex differences in cataract surgery outcomes.

Most of the patients included in the present study were employed. Employed patients reported a statistically significant improvement in QoL in the physical health, psychological health, social relationships and environment domains. The median score on the AIS was significantly higher for employed patients than for those who were unemployed. While there are many multidimensional studies on the QoL of cataract surgery patients in the literature, few of them analyze the benefits of cataract surgery in the context of the patient’s labor market status. Kara-Junior et al. found in their study that cataract surgery improves productivity at work and encourages inactive individuals to seek employment [48]. Astbury et al. stressed that it is important to provide cataract surgery patients with advice regarding return to work as part of good postoperative care [49]. The findings from a study by Finger et al. indicate that cataract surgery has a broad positive impact on the patient’s life, improves its quality and may help reduce poverty [50]. Moreover, it seems that return to work can improve cognitive function in patients after cataract surgery.

The present study including patients after cataract surgery showed a significant relationship between place of residence and scores for the social relationships domain of QoL. Patients living in urban areas reported higher scores than patients living in rural areas. The finding is consistent with the findings from a study by Partyka-Mizeracka et al. including Polish cataract surgery patients, which showed a positive correlation between the size of the place of residence and scores for the physical health, psychological health and social relationships domains of the WHOQOL-BREF [47]. Moreover, the study showed a significant relationship between education and all the domains of QoL assessed by the questionnaire. No relationship was found, however, between education and the level of satisfaction with health. Patients with higher education reported higher scores for all the domains of the WHOQOL-BREF. The study also showed that the older the patients were, the poorer their QoL was [47]. A study by Zhao et al. demonstrated an association between older age, living in a rural area and a low level of education and a more severe cognitive impairment resulting from vision loss due to cataract [44]. In the context of the Polish rural population, which is characterized by a large proportion of older individuals and has limited access to higher education due to geographical remoteness, factors such as age and level of education may contribute to differences in QoL between patients living in rural areas and those living in urban areas. These findings indicate that socioeconomic and demographic factors have a significant impact on the QoL of patients undergoing cataract surgery.

In the present study, 99% of the patients surveyed reported that they visit their ophthalmologist to monitor eye health. A number of authors have highlighted the importance of postoperative care, including the monitoring of visual outcomes and the provision of the necessary support, in ensuring optimal improvement in the patient’s QoL. Effective postoperative care can help prevent complications, reduce discomfort and promote faster healing, potentially leading to better visual outcomes and improved overall QoL [6,29,32,51,52].

As part of their study, Wu et al. developed a smartphone application for patients after cataract surgery that enables them to self-record and report their postoperative symptoms and provides an instant feedback system. A total of 79% of respondents stated that the application had improved their QoL [53]. In a study by Porela-Tiihonen et al., patients who reported postoperative ocular symptoms, such as pain and discomfort, were less satisfied with the outcome of the treatment at 12 months after cataract surgery [53]. Appropriate postoperative interventions, such as the administration of pain medication and tear substitutes, can potentially reduce some of the symptoms, which underscores the importance of monitoring the postoperative symptoms reported by patients [53]. Zuo et al. noted in their study including cataract surgery patients that high-quality nursing care can significantly accelerate the achievement of appropriate postoperative visual acuity, reduce intraocular pressure and lower the incidence of complications [53,54]. It can also improve the mental well-being and QoL of patients after cataract surgery [55].

The most recent studies in the literature on the subject analyze not only the overall QoL of cataract surgery patients but also its domains. The factors affecting QoL after cataract surgery should be analyzed from the perspective of aspects relating to the surgery itself and the hospital stay and from the perspective of the impact of the visual function improvement following cataract surgery on the patient’s functioning. It seems that the former relates mainly to short-term QoL, whereas the latter relates to long-term QoL. Regarding aspects relating to the procedure itself, advances in cataract surgery, such as the use of smaller, self-sealing incisions, made it possible to reduce the duration of the operation. This in turn has made it possible for anesthesiologists to use short-acting anesthetic agents as the standard [24,56,57]. A review of the relevant literature shows that these two factors may have an impact on QoL in all surgical patients—not only those undergoing eye surgery.

An analysis of new trends in research on the QoL of cataract surgery patients shows that the literature on cataract surgery of this decade and the previous decade not only includes a large number of geographically varied studies involving a multidimensional analysis of outcomes from cataract surgery and its impact on the patient’s functioning but also includes studies that stress that it is necessary to implement new uniform standards for cataract surgery outcome reporting, without which direct comparisons between institutions are limited [26,58,59]. Previous single- and multi-center studies have consistently suggested that a consensus on this issue can improve the quality of data, which, in the long term, will make it possible to draw conclusions based on global comparisons [26,58,59,60].

To answer the aforementioned needs, the International Consortium for Health Outcomes Measurement (ICHOM) developed, following consultations with clinicians, researchers and patient representatives, free standard sets for several conditions, including cataracts [26]. The cataract surgery standard includes two categories of mutually complementary data: traditional clinician-reported outcome measures (CROMs), such as the postoperative visual acuity and refraction data and data on complications, and patient-reported outcome measures (PROMs) tracked via the CATQUEST-9SF questionnaire [26,59]. In a study by McAlinden et al., the questionnaire was found to be the most responsive to cataract surgery of all the 16 questionnaires analyzed and thus ideal for measuring limitations in daily activities caused by cataracts [60,61]. The ICHOM standard set for cataract surgery is characterized by a consistent clinical pathway [26]. Moreover, it also takes into account treatment costs as well as case-mix variables, such as demographic factors, age, sex, baseline vision, comorbidities, type of cataract, previous cataract surgery and other previous eye surgery [59]. This compilation of data is a response to literature findings suggesting that the investigation of cataract surgery outcomes should focus on functional status and QoL, as PROMs cannot be obtained directly from the measurement of visual acuity, which does not fully reflect the patient’s visual function [60].

One study showed that the implementation of the ICHOM standard set for cataract surgery into practice at one center in Italy not only contributed to the development of a uniform evaluation system but also enabled a critical assessment of all the services provided to patients during their stay in the center, optimized medical records templates and planned further measures for improving patient outcomes [59]. However, the implementation of the standard requires the cooperation of the whole team to integrate the relevant procedures into daily practice. The findings from studies using the ICHOM standard set for cataract surgery confirm that an analysis of cataract surgery outcomes must take into account both PROMs and CROMs and that there is a statistically significant low correlation between the two types of measures, which indicates that the relationship between them is multifactorial and highlights the need for further research [60]. While the present study used validated diagnostic tools, it is not part of this emerging line of research.

Cataract surgery is currently one of the most rapidly evolving fields in ophthalmology, and advances to date have brought significant benefits in terms of improving patient QoL. Therefore, it is necessary to carry out regular research on the QoL of patients undergoing cataract surgery, as it can help improve not only procedures directly related to the treatment process but also perioperative and long-term care.

### The Study Limitations

We acknowledge that our present study has some limitations. The study did not investigate the clinical efficacy of cataract surgery, which is also likely to have a significant impact on patients’ postoperative quality of life. The study group was limited, and the single-institution design may not be fully representative of the broader Polish demographic. The advanced age of the respondents and the circumstances under which the survey was administered may have limited their ability to engage in prolonged and nuanced responses. Furthermore, many participants exhibited difficulties with sustained attention, which may have compromised the reliability of their answers. In consideration of these factors, an abbreviated and adapted version of the NEI VFQ-25 questionnaire was employed to facilitate respondent engagement. An evaluation of quality of life before cataract surgery is also missing in our study. Future research directions could include investigating the clinical efficacy of cataract surgery, including visual acuity outcomes and complication rates, to provide a more comprehensive understanding of its impact on patients’ quality of life. It should also be a multicenter study with a larger, more diverse sample size to increase the generalizability of the findings to the broader Polish population.

## 5. Conclusions

1. The respondents reported the highest QoL scores for the environment domain and the lowest QoL scores for the social relationships domain and had high levels of illness acceptance. QoL had a positive impact on illness acceptance in the patients included in the study.
2. Younger patients (aged 50 or under) reported significantly higher scores for all domains of QoL. Being employed was found to be associated with better QoL and greater illness acceptance.

## Figures and Tables

**Table 1:** QoL domain scores and AIS scores.

Parameter	Median	IQR	Min.	Max.
Physical health domain	24	23–26	4	31
Psychological health domain	22	21–25	14	27
Social relationships domain	12	11–14	6	15
Environment domain	32	29–34	17	40
AIS	34	31.5–39	8	40

SD—standard deviation, Q1—lower quartile, Q3—upper quartile, IQR—interquartile range.

**Table 2:** Correlation between QoL domain scores and the AIS score.

Parameter	AIS
	Spearman’s Correlation Coefficient
Physical health domain	r = 0.454, *p* < 0.001 *
Psychological health domain	r = 0.398, *p* < 0.001 *
Social relationships domain	r = 0.471, *p* < 0.001 *
Environment domain	r = 0.738, *p* < 0.001 *

* Statistically significant relationship (*p* < 0.05).

**Table 3:** Impact of age on QoL domains.

Parameter	Age	Median	Min.	Max.	*p*
Physical health domain	50 or under—A	26	23	28	*p* < 0.001 *
	51–60—B	23.5	17	30	A > B, D, C
	61–70—C	23	4	31	
	>70—D	23	18	30	
Psychological health domain	50 or under—A	26	22	27	*p* < 0.001 *
	51–60—B	22	18	27	A > B, C, D B > D
	61–70—C	22	14	26	
	>70—D	21	15	26	
Social relationships domain	50 or under—A	15	11	15	*p* < 0.001 *
	51–60—B	12	8	15	A > B > C, D
	61–70—C	11	7	15	
	>70—D	11	6	14	
Environment domain	50 or under—A	36	29	40	*p* < 0.001 *
	51–60—B	32	24	40	A > B > C, D
	61–70—C	31	17	37	
	>70—D	31	20	32	

*p*—Kruskal–Wallis test + post-hoc analysis (Dunn’s test), SD—standard deviation, Q1—lower quartile, Q3—upper quartile. * Statistically significant relationship (*p* < 0.05).

**Table 4:** Impact of sex on QoL domains and illness acceptance (AIS).

Variable	Female			Male			Test Result
	Me	Min.	Max.	Me	Min.	Max.	
Physical health domain	14.00	10.0	17.0	13.00	8.0	15.0	*p* = 0.150
Psychological health domain	15.00	11.0	18.0	15.00	10.0	17.0	*p* = 0.199
Social relationships domain	16.00	11.0	20.0	15.00	8.0	20.0	*p* = 0.529
Environment domain	16.00	12.0	20.0	16.00	9.0	20.0	*p* = 0.922
AIS score	34.00	8.0	40.0	34.00	11.0	40.0	*p* = 0.667

Me—median; Min.—minimum value; Max.—maximum value; *p*—level of significance.

**Table 5:** Impact of work status on QoL domains and illness acceptance (AIS).

Variable	Employed			Unemployed			Test Result
	Me	Min.	Max.	Me	Min.	Max.	
Physical health domain	14.00	10.0	16.0	13.00	8.0	17.0	*p* < 0.001
Psychological health domain	15.00	12.0	18.0	15.00	10.0	17.0	*p* = 0.006
Social relationships domain	16.50	12.0	20.0	15.00	8.0	20.0	*p* < 0.001
Environment domain	17.00	12.0	20.0	16.00	9.0	19.0	*p* < 0.001
AIS score	36.00	8.0	40.0	33.00	10.0	40.0	*p* < 0.001

Me—median; Min.—minimum value; Max.—maximum value; *p*—level of significance.

**Table 6:** Impact of place of residence on QoL domains and illness acceptance (AIS).

Variable	Urban area			Rural area			Test Result
	Me	Min.	Max.	Me	Min.	Max.	
Physical health domain	14.00	10.00	17.00	13.00	8.00	16.00	*p* = 0.116
Psychological health domain	15.00	11.00	18.00	15.00	10.00	17.00	*p* = 0.133
Social relationships domain	16.00	8.00	20.00	15.00	9.00	20.00	*p* = 0.022
Environment domain	16.00	9.00	20.00	16.00	10.00	20.00	*p* = 0.431
AIS score	35.00	8.00	40.00	33.00	18.00	40.00	*p* = 0.153

Me—median; Min.—minimum value; Max.—maximum value; *p*—level of significance.

**Table 7:** Multivariate linear regression model of quality of life in the physical health domain.

Variable		Parameter	95%CI		*p*
AIS		0.178	0.08	0.275	0.001 *
Age	50 or under	ref.			
	51–60	−1.067	−2.985	0.85	0.278
	61–70	−1.183	−3.209	0.842	0.255
	>70	0.607	−1.964	3.178	0.645
Gender	Female	ref.			
	Male	−0.91	−2.203	0.383	0.171
Work status	Employed	ref.			
	Unemployed	−2.178	−3.913	−0.444	0.016 *
Place of residence	Urban	ref.			
	Rural	−0.373	−1.858	1.112	0.624

*p*—multiple linear regression. * Statistically significant (*p* < 0.05).

**Table 8:** Multivariate linear regression model of quality of life in the psychological health domain.

Variable		Parameter	95%CI		*p*
AIS		0.128	0.056	0.2	0.001 *
Age	50 or under	ref.			
	51–60	−1.321	−2.739	0.097	0.071
	61–70	−1.759	−3.257	−0.261	0.024 *
	>70	−2.441	−4.342	−0.539	0.014 *
Gender	Female	ref.			
	Male	−0.306	−1.262	0.65	0.532
Work status	Employed	ref.			
	Unemployed	−0.04	−1.323	1.243	0.951
Place of residence	Urban	ref.			
	Rural	−0.221	−1.319	0.878	0.695

*p*—multiple linear regression. * Statistically significant (*p* < 0.05).

**Table 9:** Multivariate linear regression model of quality of life in the social relationship domain.

Variable		Parameter	95%CI		*p*
AIS		0.104	0.052	0.156	<0.001 *
Age	50 or under	ref.			
	51–60	−1.003	−2.028	0.023	0.059
	61–70	−1.813	−2.896	−0.729	0.001 *
	>70	−2.215	−3.591	−0.84	0.002 *
Gender	Female	ref.			
	Male	−0.127	−0.819	0.565	0.72
Work status	Employed	ref.			
	Unemployed	−0.526	−1.453	0.402	0.27
Place of residence	Urban	ref.			
	Rural	−0.576	−1.371	0.218	0.159

*p*—multiple linear regression. * Statistically significant (*p* < 0.05).

**Table 10:** Multivariate linear regression model of quality of life in the environment domain.

Variable		Parameter	95%CI		*p*
AIS		0.4	0.308	0.492	<0.001 *
Age	50 or under	ref.			
	51–60	−2.466	−4.279	−0.654	0.009 *
	61–70	−3.779	−5.693	−1.865	<0.001 *
	>70	−3.842	−6.272	−1.412	0.003 *
Gender	Female	ref.			
	Male	−0.175	−1.398	1.047	0.779
Work status	Employed	ref.			
	Unemployed	−1.032	−2.671	0.607	0.221
Place of residence	Urban	ref.			
	Rural	0.268	−1.136	1.671	0.709

*p*—multiple linear regression. * Statistically significant (*p* < 0.05).

## Data Availability

The data that support the findings of this study are available from the corresponding author upon reasonable request.
